# Geroprotectors and Skeletal Health: Beyond the Headlines

**DOI:** 10.3389/fcell.2022.682045

**Published:** 2022-02-09

**Authors:** Alexandra Rayson, Maya Boudiffa, Maneeha Naveed, Jon Griffin, Enrico Dall’Ara, Ilaria Bellantuono

**Affiliations:** ^1^ Healthy Lifespan Institute, Department of Oncology and Metabolism, The Medical School, Sheffield, United Kingdom; ^2^ Healthy Lifespan Institute, Department of Molecular Biology and Biotechnology, The University of Sheffield, Sheffield, United Kingdom; ^3^ Insigneo Institute for in silico Medicine, Sheffield, United Kingdom

**Keywords:** aging, senescence, osteoarthritis, osteoporosis, geroprotectors, mouse models

## Abstract

Osteoporosis and osteoarthritis are the most common age-related diseases of the musculoskeletal system. They are responsible for high level of healthcare use and are often associated with comorbidities. Mechanisms of ageing such as senescence, inflammation and autophagy are common drivers for both diseases and molecules targeting those mechanisms (geroprotectors) have potential to prevent both diseases and their co-morbidities. However, studies to test the efficacy of geroprotectors on bone and joints are scant. The limited studies available show promising results to prevent and reverse Osteoporosis-like disease. In contrast, the effects on the development of Osteoarthritis-like disease in ageing mice has been disappointing thus far. Here we review the literature and report novel data on the effect of geroprotectors for Osteoporosis and Osteoarthritis, we challenge the notion that extension of lifespan correlates with extension of healthspan in all tissues and we highlight the need for more thorough studies to test the effects of geroprotectors on skeletal health in ageing organisms.

## Introduction

In the United Kingdom, musculoskeletal disorders are responsible for approximately one third of General Practitioner consultations and for a NHS budget of nearly £5 billions/annum (Executive, 2015). In addition, an estimated 8.9 million working days were lost in 2020 for musculoskeletal disorders, accounting for 34% of all working days lost due to ill-health in the United Kingdom alone (Health and Safety Executive, 2020). As a whole, musculoskeletal disorders cause more functional limitations in the adult population in the western world than any other group of disorders (Woolf and Pfleger, 2003).

The two most frequent musculoskeletal diseases are osteoarthritis (OA) and osteoporosis (OP). OA, the most common age-related joint pathology, is characterized by cartilage degradation and inflammation in the joint, thickening of the bone plate, changes in subchondral bone and formation of osteophytes (Lotz and Caramés, 2011). Symptomatic knee OA occurs in 10% of men and 13% of women aged 60 years or older (Zhang and Jordan, 2010). The number of people affected with symptomatic OA is likely to increase due to the aging of the population and the obesity epidemic, an important risk factor driving OA (Raud et al., 2020). Patients with OA have higher levels of comorbidity compared to those of similar age without OA (Kadam et al., 2004). OA is significantly associated with other musculoskeletal diseases such as other arthropathies, synovial and tendon disorders and non-musculoskeletal comorbidities such as gastritis, intestinal diverticula and ischemic heart disease (Kadam et al., 2004). There is no effective cure for OA. Current management of OA is limited to symptoms’ alleviation (Bijlsma et al., 2011) followed by joint replacement when pharmacological management of the pain is no longer effective.

OP is characterised by significant bone loss and increased risk of fractures. Osteoporosis is defined by the World Health Organization when the mineral bone density of a person is 2.5 standard deviations below the young normal mean. It is most frequent in post-menopausal women and it affects around one in five men and one in two women over the age of 50 (Poole and Compston, 2006). Although present drugs reduce the risk of fractures, the number needed to treat (i.e., the number of patients that need to be treated for one to benefit compared with a control in a clinical trial) to prevent a fracture is >50 over 1–3 year period (Crandall et al., 2014), suggesting the need to find new more effective interventions for OP. Furthermore, 92% of patients affected with osteoporosis present other age-related comorbidities that can include cardiovascular, neurological and gastric conditions (Salive, 2013).

The frequent association of both OP and OA with co-morbidities often results in problems of polypharmacy, including increased adverse events and reduced efficacy of their treatments due to drug-drug interaction or disease-drug interactions (Van Der Heide et al., 2018). Up until now, research has focused predominantly on the identification of drugs for the maintenance of function of single tissues (i.e., bone or cartilage) or the identification of treatments for individual musculoskeletal diseases. However, as both conditions are associated with high level of comorbidities and polypharmacy, this approach is ineffective (Tinetti et al., 2012) and approaches which target clusters of diseases would be an advantage.

### Osteoarthritis and Ageing

The onset of OA is characterised by alteration in the extracellular matrix (ECM) produced by chondrocytes, which stimulates their increased proliferative response, in an attempt to restore articular cartilage (Goldring, 2000). This leads to formation of chondrocyte clusters and increased synthesis of irregular matrix components such as proteoglycans and collagen (Rothwell and Bentley, 1973). With advancing age and OA progression chondrocytes show hallmarks of ageing, such as mitochondrial dysfunction and increased oxidative stress, senescence and inflammation (Loeser et al., 2002; Grishko et al., 2009; Goldring and Otero, 2011; Loeser et al., 2016) as well as aging associated changes in autophagy (Caramés et al., 2010). This results in a reduced ability to produce ECM and an increase in catabolic processes largely mediated by proinflammatory cytokines and mediators such as metalloproteinases (Burrage et al., 2006). In turn, the ECM becomes more vulnerable to damage, leading to the onset of OA, increased cartilage degradation and disease progression. The importance of ageing in driving the disease is highlighted by the fact that aged mice show signs of cartilage degradation and develop the full osteoarthritis phenotype faster and more aggressively than young mice after destabilization of the medial meniscus or following injury (Huang et al., 2017). Therefore, targeting mechanisms of ageing may offer new opportunities for treatment for OA.

### Osteoporosis and Ageing

The adult skeleton is continuously remodelled by osteoclasts, which resorb bone, osteoblasts, which form new bone and osteocytes. Osteocytes derives from osteoblasts and are contained in the bone matrix. Through secreted factors they coordinate the activity of osteoclasts and osteoblasts in response to physical and hormonal stimuli. Osteocytes, Osteoblasts and their precursors secrete RANK-L which binds to RANK (receptor activator of nuclear factor κ-B) receptor on osteoclasts precursors, initiating their proliferation and differentiation to mature osteoclasts able to resorb bone. Osteoclast activation is inhibited by another protein known as osteoprotegerin (OPG) produced by osteocytes and osteoblasts, which acts as a decoy RANK-L and therefore competes with RANK-L for receptors. Although RANK-L and macrophage colony stimulating factor (M-CSF) are essential for osteoclastogenesis, additional cytokines such as TNF-alpha and IL-1 are likely to contribute to the regulation of osteoclast formation both in physiological and pathological condition such as oestrogen deficiency in postmenopausal women. Features of bone ageing include reduction in bone mass and bone mineral content, changes in bone shape and structure with loss of trabecular bone, thinning of cortical bone and increased porosity, enlargement of the medullary cavity, higher levels of bone marrow fat, and increase in bone turnover (Pignolo et al., 2019). At the cellular level, there is an increase in osteoclast resorption and a decrease in osteoblast bone formation, leading to a reduction in bone density and increased risk of fracture. Increased age has long been associated with reduced bone mass, which is largely thought to be due to hormonal deficiency, mainly oestrogen due to menopause. However, age-associated bone loss occurs even in individuals with normal levels of sex steroids (Riggs et al., 2008) and there is a close association between the effects of loss of oestrogen and dysregulation of mechanisms driving ageing*.* Similarly, to OA dysregulation of mechanisms of ageing such as inflammation, autophagy, increased oxidative stress and senescence have also been associated with OP (Farr and Khosla, 2019; Yin et al., 2019). Some of these mechanisms have been shown to be deficient in presence of decreased oestrogen. In an ovariectomised rat model, a significant reduction in levels of autophagy in osteocytes correlated with an increase in oxidative stress and bone loss (Yang et al., 2014). In addition, ovariectomy (OVX) resulted in significant acceleration of the epigenetic clock, the DNA methylation changes occurring with age (Stubbs et al., 2017) suggesting a close link between oestrogen deficiency and ageing, the two main drivers of OP. Therefore, ways to target mechanisms of ageing may offer new opportunities for the development of improved treatment in OP.

## Geroprotectors

Recent work has shown that it is possible to prevent or even reverse the dysregulation of oxidative stress, autophagy and the occurrence of senescence using a new class of drugs called geroprotectors. Geroprotectors are drugs that delay or reverse ageing processes and in doing so target the major risk factors for age-related diseases. They promise to promote health span of more than one organ system at the same time in animal models (Figueira et al., 2016; Bellantuono, 2018). Studies in model organisms or retrospective studies in patients show that they can ameliorate tissue dysfunction and reduce the onset and severity of many diseases [reviewed in (Morsli and Bellantuono, 2021)]. Over 200 compounds have been classified as geroprotectors, each reported to slow ageing and/or extend lifespan in a variety of organisms (geroprotectors.org).

Such drugs could have distinct advantages over present treatments in OP and offer new opportunities for OA due to the fact that they may be able to prevent both OP and OA and their co-morbidities. However, the effects of geroprotectors on skeletal health have received little attention compared to other organ systems with the assumption that these drugs will work equally well for all tissues. Here we review the evidence available to address whether geroprotectors have potential for the care of skeletal age-related diseases and their co-morbidities. We focus on drugs with a good safety profile, which have been shown to target ageing pathways, extend the lifespan and healthspan in animal models and have some evidence of improving health in humans by demonstrating protection from multiple-age-related diseases (Partridge et al., 2020) and for which there are well designed studies in animal models of OP and OA or clinical data available.

Among the most studied geroprotector is Rapamycin. It is an inhibitor of the mTOR signalling pathway, a nutrient sensing pathway closely associated with ageing and longevity (Johnson et al., 2013; Saxton and Sabatini, 2017). mTOR is a highly conserved biological pathway, encompassing two distinct complexes mTORC1 and mTORC2. The two complexes differ in composition and function, with mTORC1 having Raptor and mTORC2 containing Rictor (Saxton and Sabatini, 2017). mTORC1 is acutely inhibited by Rapamycin, whereas mTORC2 requires chronic exposure to be affected (Li et al., 2014). It targets multiple mechanisms of ageing including autophagy, oxidative stress, DNA repair (Li et al., 2014; Figueira et al., 2016). More recently Rapamycin has also been shown to inhibit the Senescence Associated Secretory Phenotype (SASP) (Wang et al., 2017), composed of pro-inflammatory and tissue remodelling factors and secreted by senescent cells. Already in clinical use as an immunosuppressor, Rapamycin has been tested extensively in animal models of ageing and age-related diseases [reviewed in (Morsli and Bellantuono, 2021)]. An analogue of Rapamycin, RAD001, has been tested in clinical trials at a substantially lower dose to elicit geroprotective effects to delay immunosenescence with excellent tolerability (Mannick et al., 2014; Mannick et al., 2018).

Metformin, used in the treatment of type 2 diabetes (T2D) (Bosi, 2009; Aroda et al., 2017), has also been extensively investigated for its additional mechanisms of action related to ageing (Valencia et al., 2017; Kulkarni et al., 2020). This includes inhibition of inflammation, reduction in DNA damage and inhibition of SASP (Algire et al., 2012; Moiseeva et al., 2013; Ashabi et al., 2015). There are multiple evidence in animal models and retrospective human studies that metformin has positive effects on multiple age-related diseases [reviewed in (Morsli and Bellantuono, 2021)]. Indeed a clinical trial, using Metformin to extend survival and reduce the incidence of multiple diseases (the TAME study) has obtained FDA approval (Barzilai et al., 2016).

Less studied but interesting in the context of OA is Acarbose (ACA), an intestinal α-glucosidase inhibitor, FDA-approved to treat diabetes and acts by inhibiting digestion of complex carbohydrates and reducing postprandial hyperglycaemia (Dinicolantonio et al., 2015). The mechanisms by which ACA leads to lifespan extension are not well understood. It is considered a calorie restriction mimetic and it has been reported to improve parameters of health, including reduced incidence of lung tumours in males mice, reduced liver degeneration in both sexes and glomerulosclerosis in female mice (Harrison et al., 2019), improved neuromuscular function in females, balance/coordination and grip strength in both sexes (Herrera et al., 2020). Age-related cardiac hypertrophy was seen only in male mice, and this male-specific ageing effect was attenuated by ACA (Herrera et al., 2020).

Similarly, less known but tested in the context of OA is 17α-estradiol (17α-E2), a naturally occurring enantiomer of 17β-estradiol (17β-E2), yet appears to be non-feminizing due to minimal activation of classical oestrogen receptors, ERα and ERβ (Stout et al., 2016). It has been shown to extend lifespan in male mice (Harrison et al., 2014), ameliorate age-associated metabolic and inflammatory dysfunction (Stout et al., 2017) and improve male glucose tolerance across much of adult life (Garratt et al., 2017). When administered in later life it maintains body weight, with larger muscle mass and fibres, increased grip strength and coordination (Garratt et al., 2019). The metabolic improvements are sex-specific and influenced by gonadal hormones (Garratt et al., 2017). Little is known on the molecular basis of 17α-E2 on lifespan and healthspan. The metabolic improvements appear to be associated with enhanced hepatic mTORC2 signalling, increased AKT activity and phosphorylation of FOXO1 (Garratt et al., 2017), increased AMPKα and reduced mTOR complex 1 activity in visceral adipose tissue (Stout et al., 2017). These latter changes were not found in liver or quadriceps muscle (Stout et al., 2017).

Of interest, particularly in the context of OA, is Glucosamine (GluN), an amino-monosaccharide derived principally from chitin, a compound found in the exoskeleton of marine invertebrate. It is a component of glycoproteins, proteoglycans and glycosaminoglycans. The main compounds containing GluN are glucosamine hydrochloride, glucosamine sulphate, N-Acetylglucosamine (GlcNAc). Those compounds have different phamarcokinetic and pharmacodynamics and seem to act through different mechanisms, which may account in part for the heterogeneity of response observed in the different studies. For example glucosamine sulphate requires a stabiliser in the form of salt and is therefore less pure than glucosamine hydrochloride, necessitating higher dosage (Owens et al., 2004). Glucosamine (sulphate/hydrochloride) have been shown to extend lifespan in *C. Elegans* and in ageing mice by mimicking similar effects to a low carbohydrate diet. Indeed, it has been shown to activate AMPK, which in turn promotes mitochondrial biogenesis, increase aminoacid transport and inhibits glycolysis (Weimer et al., 2014). GlcNAc has also been seen to promote lifespan extension in *C. Elegans* but by promoting proteasome activity and autophagy (Denzel et al., 2014). Treatment with GlcNAc showed an increase in mobility function in models of Parkinson’s and Alzhemer’s disease, suggesting effects on healthspan as well (Denzel et al., 2014). In addition, examination of abitual use of glucosamine supplements in a retrospective study showed a lower incidence of coronary heart disease and stroke (Ma et al., 2019), and a reduction in mortality due to cancer, respiratory, digestive and cardiovascular diseases (Li Z.-H. et al., 2020). However, glucosamine users were also more active and often took additional supplements compared to the control group, making it difficult to assess whether these effects were due to GluN uptake *per se*. GluN has been shown to have a good safety profile and may have promise as geroprotector (Zeng et al., 2015) but well controlled prospective preclinical and clinical studies with long duration are required to assess whether any of the GluN compounds improves health span safely.

Spermidine is also emerging for its geroprotective properties. It is a naturally occurring polyamine, and its concentration has been shown to decline with age in both males and females rat tissues and in erythrocytes (Jänne et al., 1964). Administration of spermidine extends lifespan in aged cells, *C. Elegans*, Drosophila (Eisenberg et al., 2009) and mice (Eisenberg et al., 2016) and it has been shown to improve some parameters of health. It increase B cell function in aged mice and humans (Zhang et al., 2019), cardiac function and arterial stiffness in aged mice (Eisenberg et al., 2016) and synapse ageing in drosophila (Maglione et al., 2019). Dhal salt sensitive rats fed high salt diet receiving spermidine showed reduced high blood pressure, delayed progression of heart failure and renal abnormalities seen in presence of hypertension (Eisenberg et al., 2016). In addition, administration of spermidine reduced the severity of liver lesions in a mouse model of liver cirrhosis (Yue et al., 2017) and of retinal ganglion cell death in a mouse model of optic nerve injury (Noro et al., 2015). In a community-based cohort study participants taking spermidine showed significant lower all-cause mortality (Kiechl et al., 2018). These effects seem to be the result of increased autophagy, mitophagy and mitochondrial biogenesis (Eisenberg et al., 2016). However, changes in other mechanisms associated with ageing have also been seen in presence of spermidine supplementation such as decreased histon H3 acetylation with possible consequences for gene expression (Eisenberg et al., 2009). Safety profile will require appropriate assessment in prospective studies as the results in animal models have been obtained using a wide range of doses, some of which may show toxicity when translating into humans.

Senolytics [reviewed in (Kirkland and Tchkonia, 2020)] are among the most promising geroprotectors due to their intermittent mode of administration to periodically eliminate newly formed senescent cells. This has the potential to reduce side effects and be more cost-effective. Senescent cells are characterised by irreversible cell cycle arrest and the presence of SASP. They accumulate with age and they have been shown to be causal to multiple age-associated tissue dysfunction or age-related diseases in animal models (Jeyapalan et al., 2007; Wang et al., 2009). Senolytics work by targeting specific survival pathway and selectively inducing senescent cell death (Kirkland et al., 2017). The senolytics Dasatinib, a tyrosine kinase inhibitor, and Navitoclax, a BCL2 family inhibitor, are among those most studied. They both have anti-neoplastic activity and side effects include increased risk of bleeding, immunosuppression and increased risk of infections (Wilson et al., 2010; Futosi et al., 2012; Rodriguez et al., 2012). Dasatinib has little or no senolytic activity on its own but has senolytic activity only when given in combination with Quercetin (Zhu et al., 2015). Quercetin and Fisetin (another senolytic) are flavonoids found in plants and fruits and sold as food supplements for their antioxidant activities with a good safety profile. The dose required to elicit senolytic activity is higher than that which is recommended as a food supplement and appropriate long-term safety studies are required. Several clinical trials with these drugs are ongoing.

### Geroprotectors to Target OA

*In vitro* studies on the effects of geroprotectors are limited. Rapamycin has been found to modulate chondrocytes’ survival, cell death and senescence [reviewed in (Pal et al., 2015)]. In addition, it has been shown to increase expression of autophagy genes in human and mouse chondrocytes (Zhang et al., 2015) and in bovine cartilage explants (Caramés et al., 2012b). This increase was associated with an increased expression of two major proteins of the cartilage matrix, aggrecan and type II collagene, and a decreased expression of metalloproteinases and chemokines (Zhang et al., 2015). Similarly, metformin has been shown to increase chondrocyte survival and delay senescence by increasing AMPK and reducing TORC1 signalling (Feng et al., 2020) resulting in attenuated aggrecanase activity and proteoglycan breakdown in chondrocytes grown in presence of inflammatory cytokines (Li H. et al., 2020). Senolytics Navitoclax, Fisetin and UBX0101 showed a reduction in markers of inflammation, an increase in markers of matrix deposition (e.g., glycosaminoglycans) (Jeon et al., 2017; Zheng et al., 2017; Yang et al., 2020) and an improvement in chondrocytes proliferation (Jeon et al., 2017) in human OA chondrocytes. Glucosamine has been shown to have anabolic effects by inducing the production of hyaluronic acid in human chondrocytes and synovial cells and regulate expression of inflammatory cytokines. A summary of the key *in vitro* studies is here (Henrotin et al., 2012). Spermidine has only recently been studied in the context of OA. *In vitro* it shows chondro-protective effects from oxidative damage and cell death, and anti-inflammatory properties (Silvestri et al., 2018; D’adamo et al., 2020).

*In vivo* most studies testing geroprotectors to delay or reverse the onset of OA are mainly performed in rodent models at young age following induction of OA by destabilization of the meniscus (Table 1). Administration of Rapamycin for 10 weeks at 1 mg/kg/day after transection of the medial meniscal tibial ligament and the medial collateral ligament in 8 weeks old mice decreased cartilage degradation (Caramés et al., 2012a). Similarly, intra-articular administration of rapamycin twice a week for 8 weeks in 10 weeks old mice following destabilization of the medial meniscus (DMM) or injection of hydrogel containing rapamycin in the joint in 8 weeks old DMM mice showed a reduction of OARSI score (Matsuzaki et al., 2014; Takayama et al., 2014). In all these studies rapamycin was given immediately after the DMM and prior to the establishment of damage in young animals. A similar experimental design has been used to test other geroprotectors such as Fisetin, Metformin, Spermidine or Navitoclax with similar results (Zheng et al., 2017; Li J. et al., 2020; Chen et al., 2020; Yang et al., 2020). However, the administration of Metformin (4 mg/day in drinking water, until the animals were sacrificed), was also administered 2 weeks after DMM surgery and caused partial but significant reduction in cartilage degradation, suggesting that Metformin may be beneficial even when given at very early stages of damage (Li J. et al., 2020). This has been confirmed in adult Rhesus Macaques where Metformin was administered 1 month after surgery and significantly alleviated cartilage degradation and subchondral bone thickening with a reduction in pain-related behaviour and improvement in the duration of standing and walking (Li J. et al., 2020). Similarly, testing of glucosamine in young rats showed attenuation of cartilage degradation following transection of the anterior cruciate ligament using either GluN sulphate (Wen et al., 2010) or hydrochloride (Naito et al., 2010) even when given 5 weeks post-induction of OA at high dose (Naito et al., 2010).

**TABLE 1:** Summary of studies testing geroprotectors to attenuate OA in experimental models and patients.

Geroprotector	Model	Age at the start of the experiment	Key findings (compared to controls)	Reference
Rapamycin 1 mg/kg/day i.p. Starting at the time of MMTL + MCL	Mice C57Bl/6—Transection of MMTL and MCL	8 weeks	↓Cartilage loss	Caramés et al. (2012a)
Rapamycin (10 µl of 10 µM solution) 2×/week intra-articular injection starting at the time of DMM	Mice C57Bl/6—DMM	10 weeks	↓Cartilage loss	Takayama et al. (2014)
Rapamycin (100 ng^−1^ µg) intra-articular gelatin hydrogel starting at the time of DMM	Mice C57Bl/6—DMM	8 weeks	↓Cartilage loss	Matsuzaki et al. (2014)
Rapamycin14 mg/kg/day	Mice UM-HET	Assessed at natural death	No difference in OA score	Ewart et al. (2020)
Acarbose 1,000 mg/kg/day	Mice UM-HET	Assessed at natural death	No difference in OA score	Ewart et al. (2020)
17-α-estradiol 14.4 mg/kg/day	Mice UM-HET	Assessed at natural death	No difference in OA score	Ewart et al. (2020)
Metformin Intra-gastric 200 mg/kg/day starting 3 days post-DMM Intra-articular 0.1 mmol/kg twice/week post-DMM	Mice C57Bl/6—DMM	8–10 weeks	↓Cartilage loss. ↑paw withdrawal threshold ↓weight-bearing asymmetry	Li et al. (2020a)
Metformin 205 mg/kg/day in drinking water 2 weeks prior to DMM 2 weeks post-DMM	Mice C57Bl/6—DMM	10 weeks	↓Cartilage loss ↓Synovitis ↓Osteophytes No effect on subchondral bone mass ↑paw withdrawal threshold ↑spontanous activity	Li et al. (2020b)
Metformin 51.7 mg/kg/day in drinking water 1 month post-PMM	Rhesus macaques—PMM	8.5–11.5 years	↓Cartilage loss ↓subchondral bone mass ↑standing and walking time	Li et al. (2020b)
Fisetin 20 mg/kg/day gavage immediately after DMM	Mice C57Bl/6—DMM	10 weeks	↓Cartilage loss ↓subchondral bone mass ↓Synovitis	Zheng et al. (2017)
Navitoclax 0.25, 1, 5 µM intra-articular injection at the time of DMM twice/week for 2 weeks	SD Rat—DMM	4–6 weeks	↓Cartilage loss ↓subchondral bone mass ↓osteophytes	Yang et al. (2020)
UBX0101 intra-articular (10 µl of 0.2–5 mM) 14 days post-ACLT 42 days post-ACLT	Mice C57Bl/6—ACLT	10 weeks	↓Cartilage loss ↓subchondral bone mass ↓Pain ↓osteophytes (only with d14 post-ACLT treatment)	Jeon et al. (2017)
UBX0101 intra-articular (10 µl of 1 mM) 2 weeks post-ACLT	Mice C57Bl/6—ACLT	19 months	↓Pain No change in cartilage loss, subchondral bone mass	Jeon et al. (2017)
UBX0101 intra-articular	Phase II clinical trial—OA patients	N/A	No difference in pain	Inc, (2020)
Glucosamine Sulphate oral 250 mg/kg/day starting at 5 weeks post ACLT for 10 days	Wistar Rats ACLT	N/A	↓Pain ↓Cartilage loss	Wen et al. (2010)
Glucosamine Hydrochloride oral (approx. 1,000 mg/kg/day) for 8 weeks	Wistar Rats ACLT	10 weeks	↓Cartilage loss ↓Bone erosion	Naito et al. (2010)
Glucosamine	Systematic review and meta-analysis of clinical trials	Approx. 40–70 years old (when available)	No difference observed on pain scores	(Liu et al., 2018) (Runhaar et al., 2017) (Zhu et al., 2018) (Wandel et al., 2010)
Glucosamine	Systematic review and meta-analysis of clinical trials	N/A	↓VAS pain No effect on WOMAC	(Ogata et al., 2018) (Simental-Mendía et al., 2018)
Spermidine 0.3–3–6 mM/day for 4–8 weeks	C57BL6 + ACLT	12 weeks	↓Cartilage loss ↓Osteophytes (@3–6 mM) ↓inflammation ↓MMP13 ↑Aggecan/collagenII	Chen et al. (2020)

MMTL, medial meniscotibial ligament; MCL, medial collateral ligament; DMM, destabilization of the medial meniscus, PMM, partial medial meniscectomy; ACLT, anterior cruciate ligament transection, i.p, Intra-peritoneal; SD, Sprague-Dawley; VAS, Visual Analogue Scale; WOMAC, Western Ontario and McMaster Universities Osteoarthritis Index.

The effects of geroprotectors become less effective when administered in older mice or in young mice when the disease is already well established. Intra-articular treatment with a new senolytic UBX0101 prevented OA disease in young mice. However, administered when OA was established only led to improvement in some aspects of the disease such as cartilage structure and pain, but no rescue of subchondral bone remodelling and osteophyte formation (Jeon et al., 2017). When the disease was triggered in 19-month-old mice administration of the same molecule in advance stages of the disease showed no improvement in cartilage structure despite evidence of senescent cell clearance from the articular cartilage (Jeon et al., 2017). This was associated with a lack of increased expression of prochondrogenic genes, suggesting that there may be an age-related decline in the proliferative capacities of articular chondrocytes and ability to produce matrix with age, impacting further on responses. Similarly a phase I clinical trial with UBX0101 in patients with moderate to severe OA did not show any improvement in pain score used as primary outcome (Inc, 2020).

Similarly little or no response was observed by our group and others when the effect of Acarbose, 17-α-estradiol and Rapamycin were assessed in aged UM-HET mice. UM-HET3 are produced by a cross between (BALB/cByJ × C57BL/6J) F1 mothers and (C3H/HeJ × DBA/2J) F1 fathers and have been used at the National Institute on Ageing Intervention Testing Programme (NIA-ITP) (Nadon et al., 2017).

**FIGURE 1:**
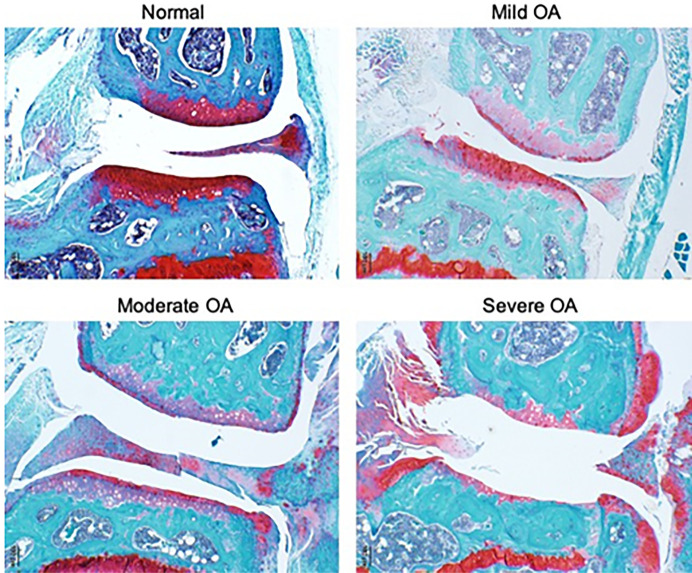
Representative images of joint pathology in UM-HET3 mice. Normal includes joints with an OARSI score of 0–.5, mild 1–2, moderate 3–4 and severe 5–6.

**FIGURE 2:**
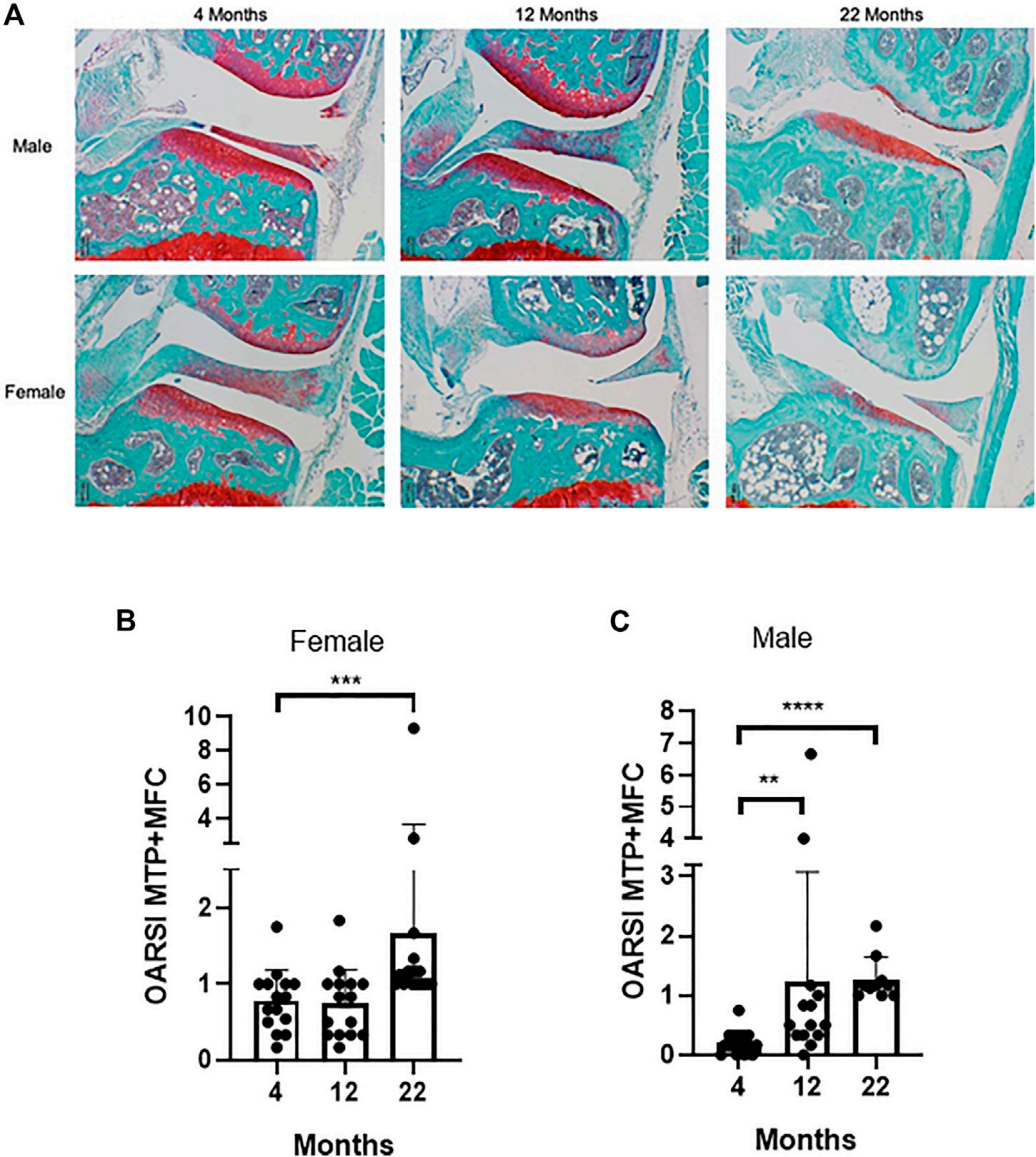
UM-HET3 mice develop joint pathology with age **(A)** Representative examples of joint pathology in male and female UM-HET3 mice at different ages; **(B)** Cartilage changes in female mice at 4 months (*n* = 16), 12 months (*n* = 15) and 22 months (*n* = 18); **(C)** Cartilage changes in male mice at 4 (*n* = 17), 12 (*n* = 14) and (*n* = 10) 22 months. Values are the mean ± SD of OARSI score for the medial tibia plateau (MTP) plus the medial femoral condyle (MFC). Data were analysed by Kruskal-Wallis test and Dunn’s multiple comparisons test, **p < .01, ***p < .001, ****p < .0001.

**FIGURE 3:**
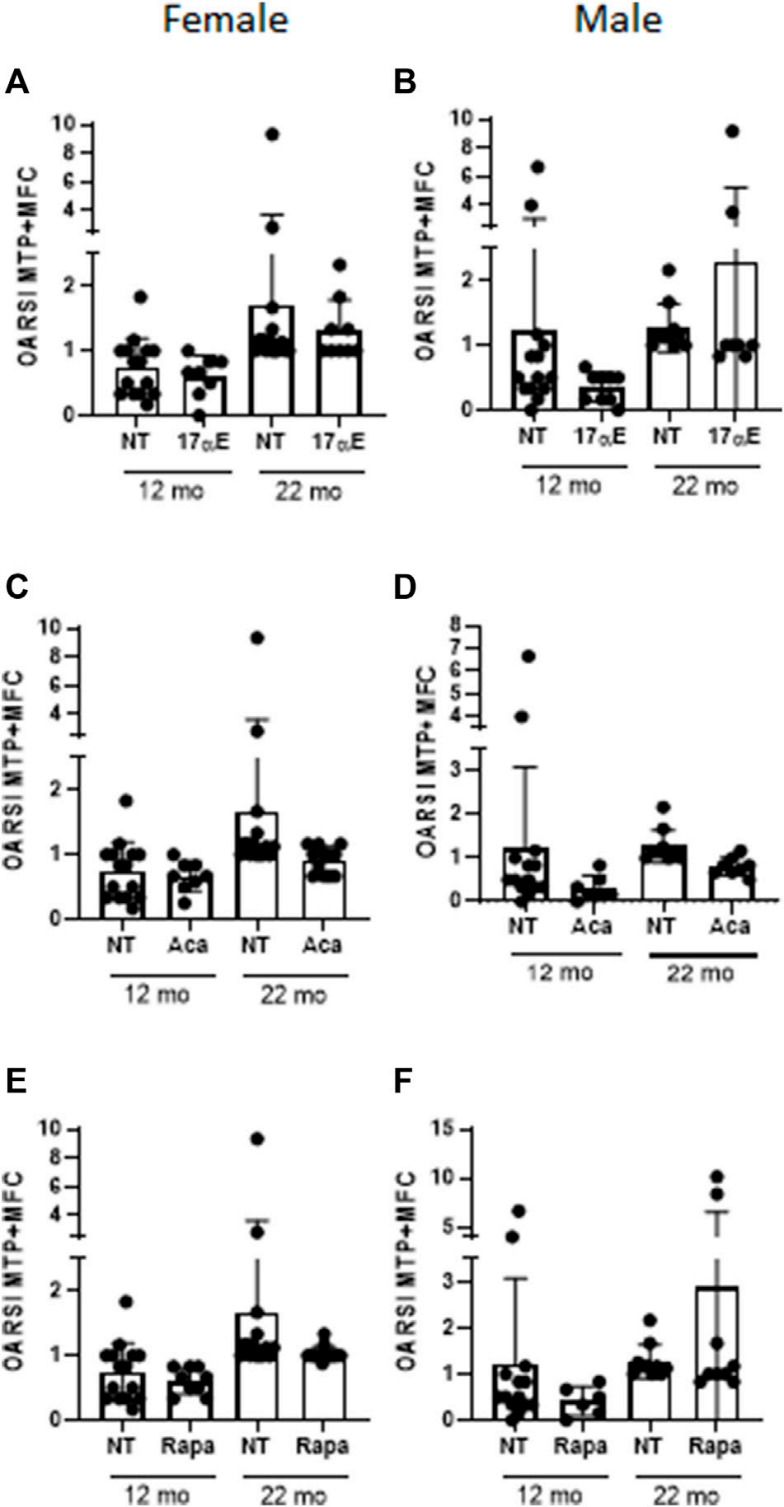
No effect on joint pathology in UMHET3 mice following treatment with 17-α-Estradiol, Acarbose and Rapamycin **(A)** Cartilage changes in female mice at 12 months (NT *n* = 15; 17 αE *n* = 8) and 22 months (NT *n* = 18; 17 αE *n* = 9) following treatment with 17-α-Estradiol (17 αE); **(B)** Cartilage changes in male mice at 12 months (NT *n* = 14; 17 αE *n* = 9) and 22 months (NT *n* = 10, 17 αE *n* = 8) following treatment with 17 αE; **(C)** Cartilage changes in female mice at 12 months (NT *n* = 15; ACA *n* = 8) and 22 months (NT *n* = 18; ACA *n* = 12) following treatment with Acarbose (ACA); **(D)** Cartilage changes in male mice at 12 months NT *n* = 14; ACA *n* = 7) and 22 months (NT *n* = 14; ACA *n* = 8) following treatment with ACA; **(E)** Cartilage changes in female mice at 12 months (NT *n* = 15; Rapa *n* = 8) and 22 months (NT *n* = 18; Rapa *n* = 12) following treatment with Rapamycin (Rapa); **(F)** Cartilage changes in male mice at 12 months (NT *n* = 14; Rapa *n* = 6) and 22 months (NT *n* = 10; Rapa *n* = 9) following treatment with Rapamycin (Rapa). Values are the mean ± SD of OARSI score for the medial tibia plateau (MTP) plus the medial femoral condyle (MFC). Data were analysed by Kruskal-Wallis test and Dunn’s multiple comparisons test, *p < 0.05, **p < 0.01, ***p < 0.001, ****p < 0.0001. NT, not treated.

All three compounds have been shown to extend lifespan in these mice, although with some sex differences (Harrison et al., 2009; Miller et al., 2011; Harrison et al., 2014; Miller et al., 2014; Strong et al., 2016; Harrison et al., 2019). Similarly to what reported in the study by (Ewart et al., 2020) our unpublished data showed a significant increase in cartilage degradation with age in these mice in both males and females (Figures 1, 2 and Supplementary Material). However, none of the treatments showed any significant improvement in cartilage degradation measured by OARSI scores in both males and females (Figures 3A–C and Supplementary Material) assessed at 12 and 22 months of age. Treatment started at 4 months of age with the exception of 17-α-E2, which started at 10 months of age when the oestrus cycle starts reducing to avoid a potential interference with sexual development (Nelson et al., 1982). Drugs were used at the same concentrations shown to elicit lifespan extension (1,000, 14, and 14.4 mg per kg of diet for acarbose, rapamycin and 17-α-E2, respectively). These data are in line with what reported by Ewart et al. (2020) when testing the effect of acarbose and 17-α-E2estradiol in the same mice using a different experimental design. In the latter, mice were collected at the time of death, whereas in our experiments the mice were culled at fixed time points, reducing some of the variability that may have arisen with the previous experimental design. It is still possible that our study was underpowered. However, even if this was the case, it indicates that any effect is very small. It is possible that these drugs may work in aged mice only in situation of challenge (e.g., following DMM surgery) and therefore they should be tested in mice following induction of OA disease in older age.

No studies are available to test the effects of Glucosamine in aged animals. However, there is an abundance of studies in patients affected by OA with very contrasting results as highlighted by meta-analysis and systematic reviews (Wandel et al., 2010; Runhaar et al., 2017; Liu et al., 2018; Ogata et al., 2018; Zhu et al., 2018). The discrepancy seems to lie in whether the trials contained biases (e.g., trials led by industry were more likely to show positive results) (Vlad et al., 2007) or the sample size (trials with over 100 patients seem to show no effect of glucosamine on pain) (Wandel et al., 2010; Runhaar et al., 2017). In addition, the formulation of glucosamine produced by Rottapharm seemed to be the most effective (Towheed et al., 2005; Vlad et al., 2007; Runhaar et al., 2017). However, it is unclear whether this is due to the formulation or to the fact that trials with the Rottapharm formulation targeted patients at early stages of disease. Whilst recent meta-analysis concluded that studies have shown modest or no efficacy of glucosamine on pain or other parameters of OA questions remain whether the treatment should be tested for longer, with a higher dose (Mccarty et al., 2019) and whether patients should be stratified based on severity of the disease and/or age, a factor that is never considered in the analysis.

Overall, these studies highlight that reducing the severity of OA in older organisms may be challenging and not sufficient on its own. These studies challenge the notion that extension of lifespan can be considered an indirect measure of health span for all tissues. They highlight the need for a comprehensive assessment of the effects of each drugs in all tissues including the skeletal tissues with natural ageing and in models of disease.

### Geroprotectors to Target OP

*In vitro* studies are primarily focused on the effects of rapamycin on osteoaclasts and osteoblasts. Based on studies using mouse, rabbit and human cells Rapamycin has been shown to reduce osteoclasts’ formation, survival and activity (Glantschnig et al., 2003; Kneissel et al., 2004; Browne et al., 2017). Effects on osteoblasts’ proliferation, survival and differentiation are inconclusive with differences in reports depending on the dose used and the species from which the cells were derived and whether they were primary or cell lines (Kneissel et al., 2004; Singha et al., 2008; Xian et al., 2012; Huang et al., 2015; Browne et al., 2017; Wu et al., 2019). This is particularly true for its effects on differentiation. For example the analogue of rapamycin Everolimus showed no effect on the osteoblast marker Alkaline phosphatase (ALP) when hMSC were induced to differentiate to the osteoblastic lineage for 7 days at 1 nM but showed a reduction in ALP expression at higher concentration of 10 and 100 nM (Browne et al., 2017). In contrast Runx2 and Osteocalcin, two other markers of osteoblasts differentiation were increased at 1 and 10 nM in the same human osteoblasts cultures but were decreased in murine cultures (Browne et al., 2017). There are many reasons for these discrepancies such as osteoblasts differentiation may proceed at different rates in human, mouse cultures and cell lines and markers of osteoblasts differentiation are dynamic, i.e., they can be upregulated and downregulated at different rates over the period of observation. Assessments of markers over multiple time points may be required to shed some light.

*In vitro* studies with other geroprotectors are very scant. Fisetin has been shown to inhibit osteoclasts’ formation and differentiation but effects on osteoblasts were not reported (Léotoing et al., 2013). Navitoclax reduced senescent cell burden but it also negatively impacted on the number of bone progenitors and osteoblasts in culture inducing apoptosis (Sharma et al., 2020). Spermidine reduced osteoclasts differentiation but did not affect their survival and had no effect on survival and differentiation of osteoblasts (Yamamoto et al., 2012). N-Acetyl glucosamine increased osteoblasts differentiation and mineralization and attenuated the negative effects of hydrogen peroxide on survival and proliferation of osteoblast (Jiang et al., 2018).

Most *in vivo* studies (summarised in Table 2) have used Rapamycin or one of its derivatives, Everolimus to test their effects on bone loss. Rapamycin and Everolimus have been shown to delay bone loss in mice in situations of challenge, i.e., in models of ovariectomy, iron load, cancer bone disease or ageing (Kneissel et al., 2004; Luo et al., 2016; Browne et al., 2017; Wu et al., 2019). These effects are primarily the result of inhibition of osteoclasts formation and activity with the exception of the study utilising the iron load model where no difference has been observed in the number of osteoclasts but an increase in ALP+ osteoblasts has been reported. A study in 24 months old rats receiving Rapamycin at 1 mg/kg/day for 12 weeks showed positive effects on osteoblasts activity with an increase in serum osteocalcin and mineral apposition rates (Luo et al., 2016). The discrepancy in recording positive effects on osteoblasts’ activity may be due to the short length of administration of Rapamycin in some of the studies (4–8 weeks). Whilst osteoclasts have shorter lifespan *in vivo* (2 weeks) osteoblasts turnover takes approximately 3 months (Manolagas, 2000) and therefore it is possible that only those studies assessing osteoblastogenesis for longer periods of time were able to detect an effect.

**TABLE 2:** Summary of *in vivo* testing of geroprotectors to attenuate bone loss in experimental models.

Geroprotector	Model	Age at the start of the experiment	Key findings (compared to controls)	Reference
Rapamycin 1 mg/kg weight/day, i.p. 12 weeks	SD rats	24 months	↑BMD ↑Trabecular BV/TV and number, thickness ↑MAR ↓N Oc and serum Tracp 5b ↑serum OCN	Luo et al. (2016)
Everolimus 0.5 mg/kg/day 1.5 mg/kg/day 3.0 mg/kg/day Gavage 4–8 weeks treatment	Wistar Rats—OVX	9 months	Attenuated cancellous bone loss ↓trabecular number No effect on cortical bone ↓N Oc No difference in cancellous bone formation rates	Kneissel et al. (2004)
Everolimus i.p 2 days post tumor injection or OVX 1 mg/kg/day for 4 weeks	Mice NMRI nude + MDA-MB-231 Mice C57BL/6 + OVX	6 weeks 9 weeks	OVX model ↑BMD ↑Trabecular BV/TV ↓N Oc Nude tumour model ↓N tumor lesions ↑BMD ↑Trabecular BV/TV ↓N Oc	Browne et al. (2017)
Rapamycin 3 mg/kg/day, i.p. for 2 months	Mice Hepcidin knockout C57Bl/6 + OVX	8 weeks	↑BMD ↑Trabecular BV/TV No difference in cortical bone ↑ N ALP + Ob No diff in N Oc	Wu et al. (2019)
Dasatinib (5 mg/kg)and Quercetin (50 mg/kg)monthly for 4 months by gavage	Mice C57Bl/6	20 months	Vertebrae ↑Trabecular BV/TV, number and thickness ↓N Oc No difference in Ob numbers, BFR, MAR Femur ↑cortical thickness ↑strength ↓endocortical N Oc ↑endocortical N Ob	Farr et al. (2017)
Fisetin 5 mg/kg/day or 50 mg/kg/day for 1 week by gavage prior to OVX followed by 5 mg/kg/day 25 mg/kg/day for 4 weeks by gavage 5 mg/kg/day 25 mg/kg/day 50 mg/kg/day for 3 weeks by gavage	Mice C57Bl/6 + OVX C57Bl/6 + LPS	8 weeks	↑BMD ↑serum OCN ↑BMD	Léotoing et al. (2013)
Navitoclax 50 mg/kg/day for 2 weeks by gavage	Mice C57Bl/6	24 months	↑Trabecular BV/TV	Sharma et al. (2020)
N-Acetyl Glucosamine 100 mg/kg/day 250 mg/kg/day for 12 weeks	Sprague-Dawley Rats	12 weeks	↑BV/TV ↑Trabecular bone area	Jiang et al. (2018)
Spermidine 0.3–3 mM/day drinking water	C57BL6 mice + OVX	8 weeks	↑BV/TV ↓N Oc	Yamamoto et al. (2012)

SD, Sprague-Dawley; i.p, intra-peritoneal; BMD, Bone mineral density; MAR, Mineral apposition rates; BFR, Bone Formation Rates; Oc, Osteoclasts; OCN, osteocalcin; Ob, Osteoblasts; ALP, Alkaline phosphatase; LPS, lypopolysaccharide.

Of interest is the fact that most studies report outcomes only in trabecular bone and do not assess cortical bone, despite both being important to confer bone strength. Kneissel et al. (2004) (Kneissel et al., 2004) reported a partial protection in trabecular bone but not in cortical bone following treatment of 9 months old rats with Everolimus for 4–8 weeks at the dose of 3 mg/kg/day. These data suggest that the effects may be limited to the trabecular bone, the more metabolic active part of the bone. Long-term studies with Rapamycin and its derivatives are required to assess its effect on osteoblastogenesis and whether both cortical and trabecular bone benefit from the intervention when exposed for prolonged periods. Careful consideration needs to be given to the dose and time of administration and the type of mTOR inhibitor as prolonged administration of Rapamycin may have side effects. Intermittent dosing has been proposed to avoid adverse events (Arriola Apelo et al., 2016). However, regimen of Rapamycin 2 mg/kg once every 5 days has been shown to inhibit mTORC1 complex but loss of glucose tolerance persisted in the same way than what was observed when given daily (Houde et al., 2010). In humans no major side effects have been seen with weekly dosing of Everolimus and this was enough to improve immune responses (Mannick et al., 2014). However, Everolimus administered at a weekly dose did not produce any difference on bone parameters (Kneissel et al., 2004), suggesting that daily dose may be required to detect effects. However, Everolimus may still be preferable to Rapamycin. Indeed when given daily it had reduced impact on glucose tolerance compared to daily Rapamycin despite being equally efficacious in inhibiting proteins of the TORC1 complex (Arriola Apelo et al., 2016).

The effect with senolytics has shown mixed results. Pharmacologic clearance of senescence cells in aged mice (20 months) treated with Dasatinib and Quercetin (DQ) for 4 months by single monthly administration showed improvement of both the trabecular and cortical bone in femur and vertebrae (Farr et al., 2017). DQ suppressed resorption by reducing osteoclast numbers and improved osteoblast numbers on the cortical bone surface but not on the trabecular bone surface (Farr et al., 2017).

When Fisetin was given to 8 weeks old mice, 1 week before OVX, an increase in trabecular bone volume fraction, thickness and number were observed 4 weeks after OVX (Léotoing et al., 2013). A similar effect was also reported when using a model of inflammation-induced bone loss by Lypopolysaccharide injection (Léotoing et al., 2013). However, it is unlikely that these effects are due to Fisetin’s senolytic activity. Very low levels of senescent cells have been reported in mice before 8 months of age (Farr et al., 2017). Studies in aged mice are required to determine whether Fisetin has senolytic effects and prevent bone loss observed with age.

Detrimental effects to trabecular bone were reported in aged male and female C57BL/6 mice (24 months old), when they were treated with the senolytic drug Navitoclax once daily for 2 weeks with signs of apoptosis on bone cells (Sharma et al., 2020). The same dose was used in the study by Chang et al. (Chang et al., 2016) to eliminate senescent cells. Indeed it showed improved proliferation and regeneration ability of hematopoietic stem cells (HSC), compatible with a reversal of HSC to a more youthful phenotype (Chang et al., 2016). However, Navitoclax was administered only for 7 days in the study by Chang et al. (Chang et al., 2016) as opposed to 14 days in the study by (Sharma et al., 2020). This may account for the toxicity observed. The toxicity of Navitoclax is well known and therefore improved regimen should be tested, particularly with the new galacto-conjugated Navitoclax, where the drug can be preferentially activated by SA-β-gal activity primarily in senescent cells (González-Gualda et al., 2020).

Studies on spermidine and Glucosamine are still in their infancy and limited to young mice. Spermidine was administered at 0.3–3 mM/day orally to 8 weeks old ovariectomised C57BL6 mice and analysed 28 days after OVX. Analysis of vertebral bone showed an increase in BV/TV associated with a decreased in the number of osteoclasts and no effects on osteoblasts (Yamamoto et al., 2012). N-Acethyl Glucosamine was administered at 250 mg/kg and 100 mg/kg/day to 12 weeks old ovarectomised Sprague-Dawley rats for 12 weeks. An increase in bone mineral density and trabecular bone area was observed. This was associated with signs of increased osteoblasts differentiation and mineralizations (Yamamoto et al., 2012). Effects on osteoclasts were no reported. Although these studies are promising, more in depth studies in aged mice are required to assess whether these agents hold promise for attenuating bone loss with age.

## Conclusion

Geroprotectors potentially have additional benefits to treat OA and OP and their co-morbidities. However, few studies focus on skeletal health despite their burden of disease. Only one study with the combination of senolytics DQ shows signs of improvement in a model of bone loss and no improvement has been demonstrated so far in aged models of OA. These studies highlight that extension of lifespan cannot be considered a surrogate marker for extension of health span in all tissues and thorough studies in aged models of OP and OA are required to assess the real benefit of geroprotectors to improve skeletal health.
