# Supplementary material for: Geroprotectors and Skeletal Health: Beyond the Headlines
Source: Front Cell Dev Biol. 2022 Feb 9;10:682045. doi: 10.3389/fcell.2022.682045 (PMC8864221; doi:10.3389/fcell.2022.682045)
Supplement: Supplementary file 2 [file DataSheet1.docx]

**Supplementary information**

**Methodology used for data presented in figure 1, 2 and 3.**

**Mice**

UM-HET3 were produced by a cross between (BALB/cByJ x C57BL/6J)F1 mothers (JAX stock #100009) and (C3H/HeJ x DBA/2J)F1 fathers (JAX stock #100004). Mice were housed at the National Institute for Ageing – Intervention testing programme (NIA-ITP), University of Michigan as previously described for the duration of the experiment [1]. These animals have received Acarbose (1000mg per kg of diet) or rapamycin (14 mg per kg of diet) from 4 months of age, or 17α-E2 (14.4 mg per kg of diet) from 10 months of age with Purina 5LG6 diet. At the appropriate time point animal were culled and knee joints were fixed and decalcified in an EDTA solution for 4 weeks, followed by paraffin embedding (coronal, medial and lateral side). Sections (3 μm) were cut from 3-4 levels (20 μm gap between levels) and stained with Safranin O-fast green to examine cartilage histopathological changes. Images were acquired using the Tissuefaxs 200 imaging system (TissueGnostics, Vienna, Austria).

**Cartilage grading**

Sections were examined for histopathological changes in articular cartilage using the Osteoarthritis Research Society International (OARSI) semi-quantitative scoring system [2]. In this system, the scores are defined as follows: 0, normal cartilage; 0.5, loss of proteoglycan with an intact cartilage surface; 1, superficial fibrillation without loss of cartilage; 2, vertical clefts and loss of surface lamina (any percentage of joint surface area); 3, vertical clefts/erosion to the calcified layer for 1–25% of the quadrant width; 4, lesion reaches the calcified cartilage for 25– 50% of the quadrant width; 5, lesion reaches the calcified cartilage for 50–75% of the quadrant width; 6, lesion reaches the calcified cartilage for over 75% of the quadrant width. The medial femoral condyle (MFC) and medial tibial plateau (MTP) for each slide were graded and scores summed. (MFC + MTP). Data were analysed by Kruskal-Wallis test and Dunn’s multiple comparisons test, with significance at p<0.05.

1. Miller, R.A., et al., *Rapamycin-mediated lifespan increase in mice is dose and sex dependent and metabolically distinct from dietary restriction.* Aging Cell, 2014. **13**(3): p. 468-77.

2. Glasson, S.S., et al., *The OARSI histopathology initiative - recommendations for histological assessments of osteoarthritis in the mouse.* Osteoarthritis Cartilage, 2010. **18 Suppl 3**: p. S17-23.
